# Lipidomics Analysis Unravels Aberrant Lipid Species and Pathways Induced by Zinc Oxide Nanoparticles in Kidney Cells

**DOI:** 10.3390/ijms25084285

**Published:** 2024-04-12

**Authors:** Boyun Kim, Gaeun Kim, Hyun Pyo Jeon, Jewon Jung

**Affiliations:** 1 Department of SmartBio, College of Life and Health Science, Kyungsung University, Busan 48434, Republic of Korea; boyunism@gmail.com (B.K.); kge1406@gmail.com (G.K.); 2 Graduate School of Chemical Safety Management, Kyungsung University, Busan 48434, Republic of Korea

**Keywords:** zinc oxide nanoparticles, lipidomics, sphingolipid, ceramide, kidney, nanotoxicity, lipid

## Abstract

Zinc oxide nanoparticles (ZnO NPs) are widely used in versatile applications, from high technology to household products. While numerous studies have examined the toxic gene profile of ZnO NPs across various tissues, the specific lipid species associated with adverse effects and potential biomarkers remain elusive. In this study, we conducted a liquid chromatography-mass spectrometry based lipidomics analysis to uncover potential lipid biomarkers in human kidney cells following treatment with ZnO NPs. Furthermore, we employed lipid pathway enrichment analysis (LIPEA) to elucidate altered lipid-related signaling pathways. Our results demonstrate that ZnO NPs induce cytotoxicity in renal epithelial cells and modulate lipid species; we identified 64 lipids with a fold change (FC) > 2 and *p* < 0.01 with corrected *p* < 0.05 in HK2 cells post-treatment with ZnO NPs. Notably, the altered lipids between control HK2 cells and those treated with ZnO NPs were associated with the sphingolipid, autophagy, and glycerophospholipid pathways. This study unveils novel potential lipid biomarkers of ZnO NP nanotoxicity, representing the first lipidomic profiling of ZnO NPs in human renal epithelial cells.

## 1. Introduction

Zinc oxide nanoparticles (ZnO NPs) are widely utilized in various industrial and biological applications, such as coating, cosmetics, paint, drug delivery systems, and biomedical engineering [1,2]. However, the expanding utilization of ZnO NPs has raised concerns regarding their potential adverse effects on human health and the environment [3,4]. It has been suggested by numerous researchers that ZnO NPs pose greater toxicity compared to other metallic oxide nanoparticles such as Al_2_O_3_ and TiO_2_, primarily due to their tendency to release ions [5]. Studies have indicated that ZnO NPs induce neurotoxicity through the generation of reactive oxygen species (ROS) by disrupting metal ion homeostasis and increasing free zinc ion levels in the cytosol [6,7]. The accumulation of zinc ions has been associated with ZnO NP-induced cytotoxicity, leading to oxidative stress and inflammation [8,9].

The ionic species Zn^2+^ holds a pivotal role within cellular signaling, serving as an indispensable regulator of protein functionality through its interactions with the sulfur moieties of cysteine residues found within cellular proteins [10]. This multifaceted ion exerts influence over a plethora of vital cellular processes, encompassing enzymatic activities, gene expression mechanisms, and the intricate pathways of signal transduction, as substantiated by prior research [11,12]. Nonetheless, it is crucial to acknowledge that an excessive influx of Zn^2+^, as observed with the introduction of ZnO NPs, can disrupt the delicate balance of cellular Zn^2+^ levels, leading to detrimental outcomes. These consequences manifest through disrupted Zn^2+^ homeostasis, resulting in cell death due to mitochondrial damage, as well as the onset of various pathophysiological conditions [13]. Among these maladies, aberrations in growth patterns, immune system dysfunction, and the onset of neurodegenerative diseases have been empirically documented [14,15,16,17]. Upon entering the biological milieu, nanoparticles, including ZnO NPs, undergo systemic circulation within the bloodstream, ultimately preferentially accumulating within vital organs such as the liver, spleen, heart, and kidney [18,19]. Given this propensity for biodistribution, it becomes imperative to assess the potential renal hazards associated with ZnO NPs, particularly in the context of their role in urinary excretion as a plausible mechanism for the elimination of excess Zn^2+^. Consequently, a comprehensive investigation into the nephrotoxic effects of ZnO NPs becomes an imperative avenue of scientific inquiry.

Metabolomics offers a valuable means to capture a momentary depiction of cellular alterations, enabling the identification of plausible metabolic mechanisms and the development of potential biomarkers for adverse effects resulting from various environmental stresses [20,21]. In recent years, numerous researchers have employed metabolomics to comprehend the intracellular toxicity mechanisms induced by nanoparticles and to identify potential biomarkers [22,23,24]. In a recent study conducted by Yan et al., a metabolomics approach was employed to uncover the pathways involved in energetic metabolism and membrane impairment in rat kidneys following the oral administration of ZnO NPs for 14 consecutive days [25]. Similarly, a prior investigation by Lee et al., utilized a nuclear magnetic resonance (NMR)-based metabolomic approach to investigate the metabolic effects of acute inhalation of ZnO particles in rat lungs [26]. The findings indicated that ZnO NPs altered the levels of metabolites associated with energy metabolism, cellular antioxidant defenses, DNA repair, and membrane structure [26]. However, the existing understanding of lipid perturbations caused by ZnO particle exposure remains limited, in contrast to the relatively well-established knowledge of hydrophilic metabolite pathways. Due to the restricted sensitivity and selectivity of NMR, the previous results only revealed changes in the lipid class containing phosphorylcholine-containing lipids (PC-CLs), which encompass numerous individual lipid species. Consequently, a more comprehensive analysis utilizing mass spectrometry (MS) is imperative to bridge the knowledge gap concerning ZnO-induced alterations in lipids and the associated toxic mechanisms.

In this study, we conducted a lipidomic investigation on human kidney cells subjected to ZnO NPs, leading to the discovery of previously unidentified lipid biomarkers and the identification of perturbed lipid-related pathways. Furthermore, we employed liquid chromatography mass spectrometry (LC/MS) to compare the lipidomic profiles between cells treated with a vehicle and those treated with ZnO nanoparticles. Lastly, we evaluated the modified lipid-related pathways using a lipid pathway enrichment analysis (LIPEA).

## 2. Results

### 2.1. ZnO NPs Induce Toxicity in Human Kidney Cells

**Figure 1:**
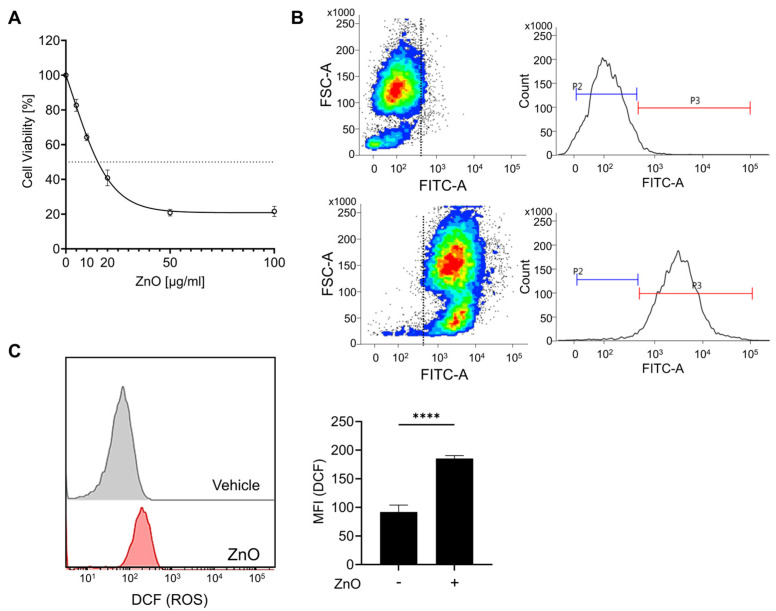
Zinc ions liberated from zinc oxide nanoparticles (ZnO NPs) initiate cell death in proximal tubule epithelial cells. (**A**) HK2 cells were subjected to varying concentrations of ZnO NPs over a 24 h period. The cytotoxic effects induced by ZnO NPs were evaluated using the MTT assay, and the determination of the IC_50_ value was conducted employing GraphPad Prism 9 software. (**B**) The intracellular concentration of Zn^2+^ was determined and quantified via flow cytometry analysis. (**C**) Levels of reactive oxygen species (ROS) subsequent to exposure to ZnO NPs were assessed using the ROS indicator DCF. Data are presented as mean ± SEM (**** *p* < 0.0001).

The physical characterization data of ZnO nanoparticles have been conducted in our previous research [27]. In brief, ZnO NPs were irregular and rod-shaped with smooth surfaces; the average size of ZnO NPs was 110 ± 41 nm [27]. The zeta potential of ZnO NPs in cell culture medium was negative (−9.7). The polydispersity index (PDI) indicates the solubility and stability of NPs in PBS and culture medium, and a PDI value lower than 0.2 is associated with a high homogeneity of the nanoparticles. The values of PDI for ZnO NPs in PBS and RPMI were 0.174 and 0.131, respectively [27]. To assess the cytotoxic impact of zinc oxide nanoparticles (ZnO NPs) on HK2 cells, sequential concentrations of ZnO NPs were administered over a 24 h period. Prior to each experimental treatment, the ZnO NPs underwent a 5 min sonication process to homogenize the particles and prevent aggregation. Exposure to ZnO NPs resulted in a notable reduction in cell viability after 24 h, with an observed IC_50_ value of approximately 20 µg/mL (Figure 1A). Subsequent experiments were conducted using a concentration of 20 µg/mL of ZnO NPs, chosen based on the previously determined IC50 value. Due to the rapid dissolution of ZnO nanoparticles into Zn^2+^, we observed the presence of liberated intracellular Zn^2+^ using FluoZin-3 AM after ZnO NP treatment. Exposure to ZnO nanoparticles resulted in the substantial release of Zn^2+^ (Figure 1B), leading to a notable decline in cell viability (Figure 1A). Xia et al. noted that the generation of reactive oxygen species (ROS) represents a primary toxicological mechanism associated with both natural and engineered nanoparticles, including ZnO NPs [28]. To validate the importance of ROS in our investigation, we assessed ROS production by detecting DCF through flow cytometry. Our findings revealed a notable increase in intracellular ROS levels upon exposure to ZnO NPs (Figure 1C). Furthermore, our previous findings demonstrated that pretreatment with NAC, aimed at eliminating excess ROS post ZnO NPs exposure, significantly mitigated the cellular damage induced by ZnO NPs [27]. These findings strongly suggest that ROS serves as the principal mediator of ZnO NPs-induced cytotoxicity.

### 2.2. Significant Alterations in Lipid Composition Are Noted Following Treatment with ZnO NPs

**Figure 2:**
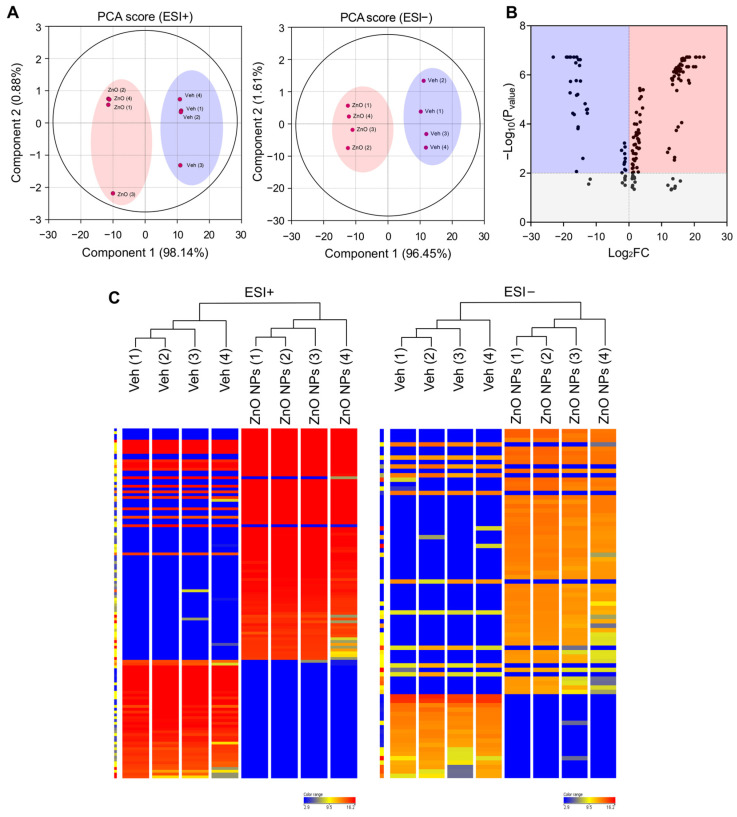
Principal component analysis (PCA) plots and hierarchical clustering analysis for lipidomic data from ZnO NP-treated HK2 cells in the negative and positive ion modes of UPLC/Q-TOF-MS. (**A**) PCA plot for HK2 cell samples between ZnO NPs treatment group and control group in positive ion mode (ESI+) and negative ion mode (ESI−). (**B**) Volcano plot illustrating metabolomics data. The x-axis denotes the mean fold-change ratio (log_2_ scale) in the relative abundance of metabolites between two samples. The y-axis indicates the statistically significant *p*-value associated with the fold-change ratio for each metabolite. (**C**) Hierarchical clustering heatmap of lipid species data for the ZnO NPs treatment group and the control group. Levels of normalized peak area are shown on the color scale, with numbers indicating the fold difference from the mean.

Lipids constitute a varied group of compounds with a wide array of structural and signaling roles. Previously, our research has concentrated on understanding the toxicity mechanisms associated with ZnO NPs. To ascertain alterations in lipid composition induced by ZnO NPs, we initially conducted untargeted lipidomics to assess their impact on HK2 cells. To compare the lipidomics data response to ZnO NPs, a principal component analysis (PCA) was carried out using total Log_2_FC datasets without threshold restrictions. Although higher dispersion was shown in ZnO NP-treated cells than in vehicle treated cells, the metabolic profile in the ZnO NPs treatment group was clearly different from that of the control exposure group (Figure 2A), indicating that endogenous metabolite levels in the treated group have changed significantly compared to the control group. To examine alterations in lipid species levels, univariate statistical analysis was utilized to identify significant differences in lipidome profiles between cells treated with ZnO NPs and control cells. Figure 2B indicates the fold changes and corresponding *p*-values of the detected lipids. The x-axis represents the log_2_ fold change in relative abundance of each species in ZnO NP-treated cells compared to untreated control cells, while the y-axis depicts the log_10_*p*-value. Volcano plot analysis revealed 170 differential metabolites between ZnO NPs and control groups (Figure 2B; Table S1). Subsequently, this dataset was further scrutinized to pinpoint the most pronounced lipid changes, employing statistical methods to identify species exhibiting significant alterations at a 95% confidence level. The alteration of lipids in the ZnO-NP-treated cells was visualized by heatmaps (Figure 2C). The heatmap visualizes the relative increase (red) or decrease (blue) of lipids in each group of samples. 124 lipids were significantly upregulated, and 46 lipids were significantly downregulated in the ZnO NPs group compared to the control group.

### 2.3. Lipid Biomarkers of ZnO NP Treatment

Potential lipid biomarkers in the kidney cells were extracted based on statistical analyses, with the parameters of the altered lipids satisfying the criteria |Log_2_FC| > 1 and *p* < 0.05. In brief, a total of 64 compounds showed alterations after treatment with ZnO NPs. In ZnO NP-treated cells, 50 and 14 compounds were upregulated and downregulated, respectively, compared to the vehicle-treated cells (Table 1). We classified altered lipids according to their lipid classes. A total of five lipid classes, i.e., glycerolipids, sphingolipids, glycerophospholipids, fatty acyls, and prenol lipids, were identified in ZnO NP-treated cells (Table 1). Our lipidomic analysis strongly emphasized the enrichment of phosphatidylethanolamine (PE) in glycerophospholipids and ceramide in sphingolipids. Specifically, every sphingolipid species accumulated, including SM and ceramides in the ZnO NPs group. Ceramides, sphingolipids that mediate cell death and have been associated with autophagy induction [29], were examined in our study. Our findings indicate a notable accumulation of ceramides, particularly species C18:0 and C18:1, subsequent to ZnO NP treatment.

### 2.4. Different Lipid Species Are Associated with Sphingolipid Metabolism

**Figure 3:**
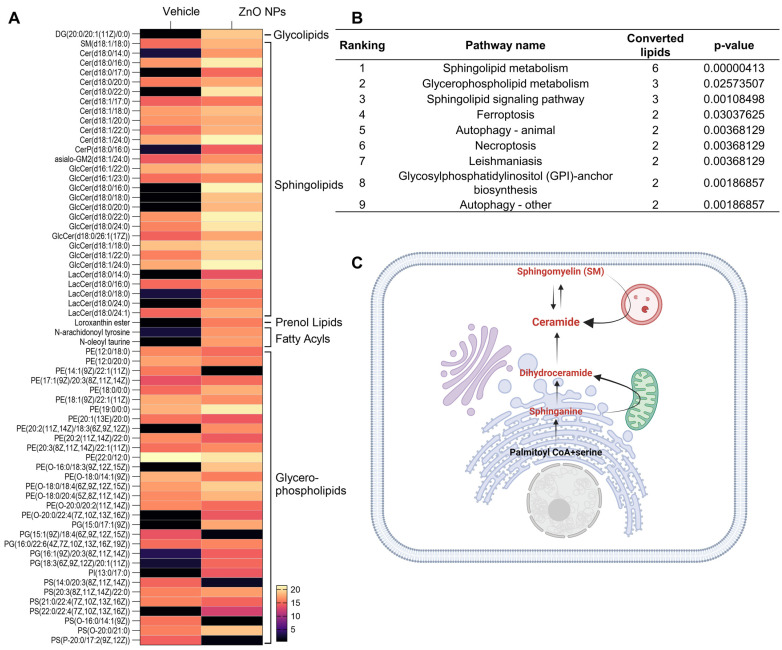
Different lipid species are associated with metabolism. (**A**) Heatmap with abundance of the interested lipids from vehicle and ZnO NPs-treated groups (listed in Table 1). (**B**) Pathway enrichment analysis of lipid metabolites by LIPEA. Results include sphingolipid metabolism, glycerophospholipid metabolism, the sphingolipid signaling pathway, ferroptosis, autophagy, necroptosis, leishmaniasis, and glycosylphosphatidylinositol anchor biosynthesis. (**C**) overview of ceramide production by the sphingolipid signaling pathway, manually curated from ZnO NPs treatment. The metabolites participating in reactions are represented in red.

To investigate the lipid alteration-related signal pathway, we carried out a LIPEA signal pathway analysis based on the KEGG database source for overexpression and pathway topology analysis. Lipid species altered by ZnO NPs exposure were presented as a heatmap (Figure 3A). Different lipid species were analyzed using LIPEA *p*-values corrected to less than 0.05, and finally, nine lipid signal pathways were found in ZnO NP-treated cells (Figure 3B). As we expected, based on the changes in lipid composition, sphingolipid metabolism, sphingolipid signaling pathways, and glycerophospholipid pathways, ZnO NP-treatment was highly ranked. In this study, the ceramide synthase pathway was manually curated, leading to the identification of a set of lipid metabolite reactions following treatment with ZnO NPs. These reactions encompass both the de novo and sphingomyelin pathways of ceramide production (Figure 3C).

### 2.5. Increase in Ceramide Levels and Induction of Cell Death by ZnO NP Treatment

**Figure 4:**
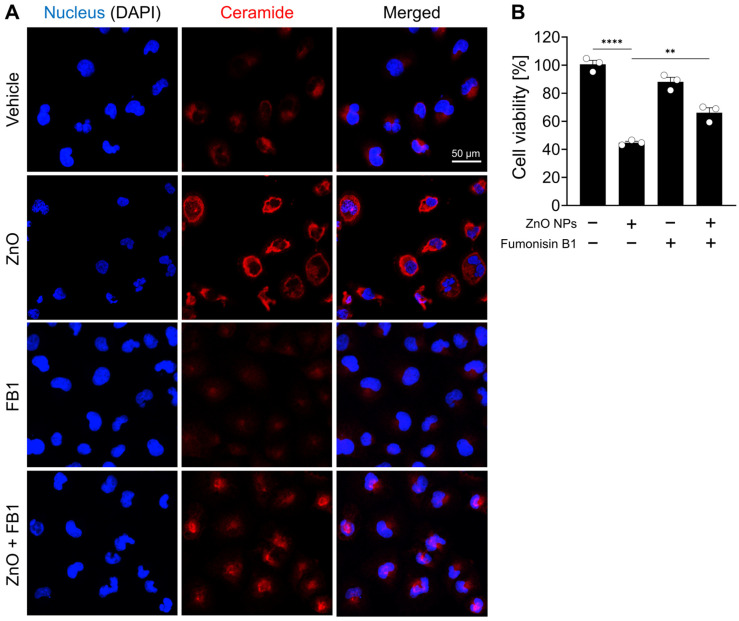
ZnO NPs increase ceramide levels and induce cell death from ceramide biosynthesis in HK2 cells. (**A**) Increased levels of ceramide in specifically HK2 cells with ZnO NPs treatment. The ZnO-induced ceramide level was decreased by fumonisin B1 (FB1). (**B**) Cell viability recovered by 20 µM fumonisin B1 was determined by MTT assay. All data are represented as the mean ± SEM. ** *p* < 0.01, **** *p* < 0.0001.

In support of the observed increase in ceramide species subsequent to ZnO NP treatment as unveiled by lipid profiling analysis, a comparative assessment of ceramide immunoreactivity was performed between ZnO NP-treated cells and their respective controls. Immunofluorescent labeling confirmed a significant elevation in ceramide levels within the ZnO-treated cells compared to the untreated control cells (Figure 4A). To investigate the role of ceramide species in the cell death induced by ZnO NPs treatment, HK2 cells were treated with fumonisin B1, an inhibitor of ceramide synthase. We confirmed fumonisin B1 treatment effectively inhibited ceramide biosynthesis induced by ZnO NP exposure, and subsequently, the cellular distribution of ceramide was restored to levels similar to those observed in the vehicle-treated group (Figure 4A). Upon exposure to ZnO NPs, the survival rate of cells was approximately 50%, whereas co-treatment with both ZnO NPs and fumonisin increased cell viability to 69% (Figure 4B). These results suggest that the heightened levels of ceramide induced by ZnO NP exposure contribute to cell death in HK2 cells.

## 3. Discussion

In the present study, we investigated the effect of ZnO NPs on lipid homeostasis in human kidney cells by using lipidomics techniques to search for biomarkers that may play a key role in inducing nephrotoxicity. Exposure to the ZnO NPs caused more prominent alterations in the lipid metabolic pathways associated with sphingolipid metabolism and glycerophospholipid metabolism compared to the control administered.

The lipidomic analyses revealed significant alterations in the glycerophospholipid metabolic pathway of HK2 cells following exposure to ZnO NPs, particularly in the PE, PS, and PG species. Among the detected changes, a total of 18 PEs, 5 PGs, and 7 PSs exhibited significant modifications in the ZnO NPs-treated group. Of these, 9 PE subclasses (50%), 4 PGs (80%), and 4 PSs (57.14%) were upregulated, indicating disruption in the homeostasis of these lipid classes within the glycerophospholipid metabolic pathway. Such disturbances are suggestive of alterations in membrane lipid composition, which may ultimately impact the physical properties and functional integrity of cellular membranes, potentially leading to apoptosis and inflammation [30,31]. In healthy mammalian cells, the plasma lipid membrane typically comprises approximately 45–55% phosphatidylcholine (PC), 15–25% phosphatidylethanolamine (PE), and 5–10% phosphatidylserine (PS). PC exhibits a uniform distribution across the cell membrane, whereas PS and PE are predominantly located on the inner leaflet rather than the outer leaflet of the plasma membrane [32]. However, during various pathological conditions such as apoptosis, thrombosis, and tumor vasculature, PS and PE are known to undergo translocation from the inner to the outer membrane leaflet, rendering them noteworthy targets for cell death processes including apoptosis and ferroptosis [33]. Pathway analysis results show that ferroptosis is induced as a cell death mechanism in cells treated with ZnO NPs. Among several membrane phospholipids, arachidonic acid-containing phosphatidylethanolamine (PE-AA) is known to be a major target of lipid peroxidation that induces ferroptosis [34], and two species among the lipid metabolites we discovered were confirmed to be PE-AA-related species in the ferroptosis pathway.

Our findings reveal an upregulation of ceramide and sphingomyelin in the ZnO NPs group, both integral components of the sphingolipid metabolic pathway. Ceramides and ceramide-derived sphingolipids serve as structural constituents of cell membranes linked to oxidative stress and inflammation, potentially contributing to liver and renal toxicity. Inflammation and an excess of saturated fatty acids prompt the continual synthesis of new ceramides [35]. Notably, our results demonstrate a significant increase in 29 ceramide subclasses within the ZnO NPs group compared to the control group. The heightened ceramide levels subsequent to ZnO NPs administration may induce autophagy-mediated cell death in renal failure [27]. Ceramide is implicated in the regulation of cell growth arrest and the induction of cell death [36]. It plays a well-established role in initiating programmed cell death in response to various stimuli, such as growth factor withdrawal, death receptor activation, hypoxia, and exposure to chemotherapeutic agents [37,38]. Although numerous studies have confirmed the significant role of ceramide in mediating lethal autophagy, the precise underlying mechanisms remain incompletely understood. An initial report by Dbaibo et al. demonstrated that arsenic trioxide (As_2_O_3_) induces the accumulation of cytotoxic levels of ceramide in human leukemia cells by promoting de novo ceramide synthesis [39]. Qian et al. further illustrated that As_2_O_3_ triggers not only apoptosis but also autophagic cell death in leukemia cell lines, with the latter being attributed to the upregulation of Beclin-1 protein and effectively prevented by the autophagy inhibitor [40]. In a study on malignant glioma cells, ceramide was found to induce autophagic cell death, as evidenced by the presence of autophagic vacuoles, acidic vesicular organelles, and LC3B-II lipidation [41]. In human leukemia cells (HL-60) and Chinese hamster ovary cells (CHO), ceramide-activated protein phosphatases (Cer-CAPPs) were found to exert inhibitory effects on the Akt-mTOR pathway, promoting autophagy and inducing autophagy-mediated cell death. Conversely, the S1P-S1P3 signaling pathway activated the Akt-mTOR pathway, counteracting autophagy and suppressing lethal autophagy [42]. Our previous research revealed that HK2 cells, human renal cells exposed to ZnO NPs, undergo cell death through autophagy [27]. Through the lipidomics signaling pathway analysis conducted in this study, we have additionally confirmed that ZnO NPs induce pathways such as autophagy and ferroptosis.

Ceramides are abundantly present in the kidney and play crucial roles in regulating various cellular processes [43]. Previous research has implicated ceramides in the pathogenesis of acute kidney injury induced by ischemic reperfusion, toxic insults, and oxidative stress [44]. In the normal mouse kidney cortex, specific ceramide species, including C24, C22, and C16, have been identified [45]. Ischemia/reperfusion or nephrotoxic injury leads to a transient reduction in renal ceramide levels, followed by a 2–3-fold increase in ceramide concentrations [46]. The ceramide synthase inhibitor fumonisin B1 attenuates hypoxia-reoxygenation or radiocontrast-induced renal tubular epithelial cell injury, suggesting that increased ceramide synthase activity contributes to elevated ceramide generation, ultimately leading to apoptotic changes in renal epithelial cells [46,47]. In this study, we observed an elevation in ceramide levels in HK2 cells exposed to ZnO NPs compared to the control group through fluorescent staining. Cell death induced by ZnO NP exposure was determined to initiate lethal autophagy processes via ceramide synthesis. The restoration of cell death was observed upon treatment with the ceramide synthesis inhibitor. In the future, it will be imperative to conduct research aimed at elucidating the mechanisms underlying the cellular homeostasis of individual species of ceramide in the induction of renal fibrosis.

In line with our current findings, Chavez Soria et al. demonstrated that treatment with copper oxide nanoparticles (CuO NPs) for 24 h led to a significant increase in the accumulation of ceramides, including C18:0, C18:1, and C22:0, in the colorectal cancer cell line HCT-116 [22]. Interestingly, CuO NPs did not induce any alterations in the composition of fatty acids and PE. Conversely, treatment with ZnO NPs for the same duration resulted in a notable upregulation not only of ceramides but also of PE species in our current findings. Of particular interest, our lipidomics data revealed that ZnO NPs triggered the accumulation of N-oleoyl taurine, which is a member of the fatty acyl superclass. This compound, recognized as *N*-acyl taurine, is commonly found in the central nervous system and kidneys, where it serves as an endogenous lipid messenger capable of activating transient receptor potential (TRP) calcium channels [48,49]. Notably, in cancer research, *N*-acyl taurine has been identified as an anticancer molecule, demonstrating the ability to induce cell cycle arrest in the human prostate adenocarcinoma cell line [50]. Moreover, the accumulation of *N*-acyl taurine in conjunction with acylcarnitine has also been implicated in the dysfunction of β cells by increasing calcium flux, contributing to the development of type 2 diabetes [51,52]. Building upon our previous research, which demonstrated that exposure to ZnO NPs induces autophagic cell death via lysosomal TRPML1 (*Mucolipin 1*) activation in HK2 cells [27], we propose that ZnO NP-induced *N*-acyl taurine may play a role in TRPML1 activation, leading to an increase in cytosolic Zn^2+^ levels through enhanced lysosomal activity. However, it is noteworthy that *N*-acyl taurine is closely associated with TRPV (Vanilloid) channels primarily located on cell membranes [48,53]. Thus, further investigation is warranted to elucidate the relationship between *N*-acyl taurine and TRP (Mucolipin) channels in the context of ZnO NPs. Moreover, the concurrent stimulation of ceramide, PE, and *N*-acyl taurine could potentially serve as markers for ZnO NP-induced cytotoxicity in kidney cells. Additional studies are required to comprehensively understand the intricate mechanisms underlying the cytotoxic effects of ZnO NPs and their implications for cellular homeostasis.

Given that this study only examined the HK2 cell line, it cannot fully capture the elaborate mechanisms of human kidney damage caused by ZnO NP exposure. Nevertheless, if we apply these findings to the human kidney organoid systems or mouse injury models, we may expect more practical and clinically relevant outcomes.

## 4. Materials and Methods

### 4.1. Reagents

Zinc oxide nanoparticles (ZnO NPs) with a diameter less than 100 nm were dispersed in phosphate-buffered saline (PBS) and subjected to ultrasonication for 5 min to prevent aggregation prior to cell treatment. The ZnO NP was then prepared in the culture medium at various concentrations. ZnO NP and 2,7-Dichlorofluorescein (DCF) were purchased from Sigma-Aldrich (St. Louis, MO, USA). FluoZin™-3, AM was purchased from Thermo Fisher Scientific (Carlsbad, CA, USA).

### 4.2. Cell Culture

HK2 cells were obtained from the Korean Cell Line Bank (Seoul, Republic of Korea). The HK2 cells were grown in RPMI-1640 (Welgene, Republic of Korea) supplemented with 4.5 g/L D-glucose, 2 mM L-glutamine, 10 mM HEPES, 1 mM sodium pyruvate, 1.5 g/L sodium bicarbonate, 10% fetal bovine serum (Gibco, New York, NY, USA), 100 U/mL penicillin, and 100 g/mL streptomycin (Gibco) under an atmosphere of humidified air containing 5% CO_2_ at 37 °C.

### 4.3. Cell Viability

To determine the toxicity of the ZnO NPs, HK2 cells in RPMI 1640 medium supplemented with 10% FBS were seeded into a 96-well culture plate (1 × 10^4^ cells/200 µL/well) in the presence of increasing concentrations of ZnO NPs (0, 1, 2, 5, and 10 µg/mL). The HK2 cells were incubated for 2 days at 37 °C in the presence of CO_2_. At the end of the incubation period, 3-(4,5-dimethylthiazol-2-yl)-2,5-diphenyltetrazolium bromide (MTT) assay was conducted to determine the viability of the HK2 cell. The cells following exposure to ZnO NPs for 24 h were incubated with 2 mg/mL MTT at 37 °C for 3 h in the dark. After removing the previous incubated medium, dimethyl sulfoxide (DMSO) was added to dissolve formazan transformed by live cells. Absorbance was measured at 540 nm by a microplate reader (SpectraMax^®^ ABS, Molecular Devices, San Jose, CA, USA).

### 4.4. Measurement of Reactive Oxygen Species (ROS)

DCF was employed for the quantification of reactive oxygen species (ROS) following the administration of ZnO NPs in HK2 cells. Subsequent to the exposure to ZnO NPs at a concentration of 20 µg/mL, HK2 cells were subjected to incubation with 2.5 µM of DCF at 37 °C for 30 min in the absence of light. The relative fluorescence intensity of DCF was assessed using a BD FACS™ Universal Loader (BD Biosciences, San Jose, CA, USA).

### 4.5. Measurement of Intracellular Zn^2+^ Level

The FluoZin™-3, AM probe was employed for the assessment of intracellular Zn^2+^ levels. HK2 cells, subjected to ZnO NPs for a duration of 24 h, were subjected to incubation with 100 nM FluoZin™-3, AM at 37 °C for a duration of 30 min in the absence of light. The relative fluorescence intensity emanating from FluoZin-3 was subsequently measured and quantified using a BD FACS™ Universal Loader (BD Biosciences, San Jose, CA, USA).

### 4.6. Lipid Extraction

Cells were washed twice with an ice-cold PBS solution. For the lipid extraction, the cells were treated with 400 µL of methanol and collected by scrapping. The collected cells were homogenized using a sterile pestle on ice for 30 s, and 200 µL chloroform was added. Sonication in an ultrasonic bath was performed for 30 s with a subsequent resting period on ice for 30 s, totaling five cycles over 5 min. Additionally, 200 µL of chloroform was further added, followed by the addition of 360 µL of water. After vortexing, the samples were centrifuged at 16,000× *g* at 4 °C for 5 min. The lower phase was transferred to glass tubes using a syringe with a needle. The transferred samples were then dried in a vacuum concentrator and stored at −80 °C until further use.

### 4.7. Lipid Analysis Using UPLC/Q-TOF-MS

The dried lipids were dissolved in 150 µL of methanol/chloroform (1:1, *v*/*v*). We employed an ultra-performance liquid chromatograph quadrupole time-of-flight mass spectrometer (UPLC/Q-TOF-MS, Agilent Technologies, Santa Clara, CA, USA; Metabolomics Research Center for Functional Materials, Kyungsung University, Busan, Republic of Korea) equipped with an electrospray ion source (ESI) for the analysis. Chromatographic separation was achieved using a ZORBAX Eclipse Plus C18 Column (95 Å, 1.8 µm, 2.1 mm × 100 mm, Agilent Technologies), with the column temperature maintained at 50 °C. A binary mobile phase system was utilized: mobile phase A consisting of water/methanol (90:10) with 10 mM ammonium acetate, and mobile phase B consisting of acetonitrile/methanol/isopropanol (20:20:60) with 10 mM ammonium acetate. Gradient elution with a flow rate of 0.3 mL/min was conducted as follows: 0 min, 55% B; 5 min, 57% B; 25 min, 100% B; 27 min, 100% B; 27.1 min, 55% B; 30 min, 55% B. For mass spectrometry, an Agilent 6545 Q-TOF (Agilent Technologies) equipped with positive and negative electrospray ionization (ESI) sources was set as follows: capillary voltage 4 kV, fragmentor voltage 160 V, gas temperature 250 °C, drying gas 10 L/min, maximum pressure of nebulizer with 35 psi, sheath gas temperature 300 °C, sheath gas flow 12 L/min, and RF voltage 750 V.

### 4.8. Lipidomic Data Processing

The Mass Profiler Professional software 15.0 (Agilent Technologies, USA) was used for the visualization, processing, and interpretation of multidimensional LC/MS data. To ensure comparability, the data underwent normalization using total ion intensity. Statistical analyses were conducted using multivariate methods, specifically principal component analysis. The student *t*-test was utilized to compare the peak height intensity of distinct lipid metabolites between normal cells and ZnO NP-treated cells. Lipids exhibiting significant differential expression were identified based on criteria of |Log_2_FC| > 1 and *p* < 0.05, indicating substantial changes in lipid levels between the two groups.

### 4.9. Pathway Analysis

In order to conduct pathway analysis, a Lipid Pathway Enrichment Analysis (LIPEA) approach was employed, utilizing a comprehensive database source such as the Kyoto Encyclopedia of Genes and Genomes (KEGG). This allowed for the visualization of pertinent pathways associated with potential lipid biomarkers.

### 4.10. Immunostaining and Confocal Microscope

After exposing HK2 cells to ZnO NPs for 24 h, they were fixed with a solution of 4% paraformaldehyde in PBS for 20 min at room temperature. Following fixation, the slides were treated with a blocking solution containing 5% normal goat serum in PBS for 1 h at room temperature. Subsequently, the cells were subjected to an overnight incubation at 4 °C with primary mouse anti-ceramide antibody (diluted at a ratio of 1:50 in PBS, Enzo Life Sciences, Farmingdale, NY, USA), followed by a 1 h incubation at room temperature with anti-mouse Alexa 560-conjugated antibody (diluted at a ratio of 1:1000 in PBS, Thermo Fisher Scientific). DAPI staining for nuclei was performed for 1 h at room temperature. The images were captured using a confocal imaging system (A1Rsi+, Nikon Instruments, Tokyo, Japan) equipped with a 40× oil immersion lens.

### 4.11. Statistical Analysis

Data were analyzed using GraphPad Prism 10 (San Diego, CA, USA) and expressed as mean ± standard error of mean (SEM) from a minimum of three independent experiments. The normal distribution was assessed using the Shapiro-Wilk test. For data that followed a normal distribution, we employed the unpaired *t*-test to compare two groups and one-way analysis of variance (ANOVA) to compare three or more categorical groups. Post-hoc analysis following a significant difference detected by one-way ANOVA was conducted using Tukey’s test.

## 5. Conclusions

In this investigation, we employed a lipidomics approach to elucidate the impact of zinc oxide nanoparticles (ZnO NPs) on a human kidney cell line (HK2). We hypothesized that alterations in lipid composition could elucidate the mechanisms underlying the toxicity of these nanoparticles and offer metabolite markers indicative of ZnO NP-induced toxicity. Furthermore, untargeted lipidomics analysis in HK2 cells exposed to ZnO NPs revealed shifts in metabolite composition, notably the accumulation of phosphatidylethanolamines (PE), phosphoglycerides (PGs), and ceramides. These findings, combined with our previous research [27], strongly indicate that ZnO NP treatment triggers autophagy and cell death through mechanisms that are independent of caspase activity but reliant on the production of ceramide species. These outcomes underscore the complexity of the mode of action of ZnO NPs, which appears to vary across different tissue cell lines. Nevertheless, further investigations are warranted to fully elucidate the mechanisms underlying the toxicity and cellular uptake of ZnO NPs in mammalian cells.

## Figures and Tables

**Table 1:** Untargeted lipidomic profiles altered by ZnO NPs exposure in HK2 cells.

Superclass	Lipid (Identification)	Observed *m*/*z*	*p* (Corrected)	Abundance		Log FC
				[veh]	[ZnO]	
Glycerolipids	DG(20:0/20:1(11Z)/0:0)	678.6162	7.4 × 10^−7^	0.536	19.035	18.499
Sphingolipids	SM(d18:1/18:0)	730.5989	3.3 × 10^−3^	14.996	18.123	3.127
	Cer(d18:0/14:0)	511.4964	2.5 × 10^−2^	2.860	16.907	14.047
	Cer(d18:0/16:0)	539.9166	2.3 × 10^−5^	16.872	20.649	14.858
	Cer(d18:0/17:0)	553.5434	1.6 × 10^−7^	0.633	14.837	14.205
	Cer(d18:0/20:0)	595.5903	1.8 × 10^−3^	15.566	17.523	1.958
	Cer(d18:0/22:0)	623.6216	1.4 × 10^−7^	0.633	20.380	19.748
	Cer(d18:1/17:0)	551.5277	3.4 × 10^−2^	14.449	15.514	1.065
	Cer(d18:1/18:0)	565.5434	9.3 × 10^−3^	17.203	18.558	1.355
	Cer(d18:1/20:0)	593.5747	3.1 × 10^−2^	16.762	17.815	1.052
	Cer(d18:1/22:0)	621.6060	2.9 × 10^−4^	14.878	18.000	3.123
	Cer(d18:1/24:0)	649.6373	4.1 × 10^−5^	17.798	21.086	3.289
	CerP(d18:0/16:0)	619.4941	1.7 × 10^−2^	2.382	14.299	11.917
	asialo-GM2(d18:1/24:0)	1176.8223	1.2 × 10^−3^	14.195	16.616	14.484
	GlcCer(d16:1/22:0)	755.6275	2.0 × 10^−2^	17.860	19.132	1.272
	GlcCer(d16:1/23:0)	769.6432	3.6 × 10^−2^	15.137	16.312	1.175
	GlcCer(d18:0/16:0)	701.5806	1.4 × 10^−7^	0.633	21.054	20.421
	GlcCer(d18:0/18:0)	729.6119	1.4 × 10^−7^	0.633	18.707	18.075
	GlcCer(d18:0/20:0)	757.6432	1.4 × 10^−7^	0.633	18.353	17.720
	GlcCer(d18:0/22:0)	785.6745	2.8 × 10^−5^	16.733	20.923	4.190
	GlcCer(d18:0/24:0)	813.7058	9.5 × 10^−6^	16.018	20.384	4.366
	GlcCer(d18:0/26:1(17Z))	839.7214	4.8 × 10^−4^	14.536	17.495	2.960
	GlcCer(d18:1/18:0)	727.5962	3.6 × 10^−2^	18.700	19.804	1.103
	GlcCer(d18:1/22:0)	783.6588	1.1 × 10^−4^	15.857	19.295	1.072
	GlcCer(d18:1/24:0)	811.6901	7.9 × 10^−5^	17.680	21.039	3.360
	LacCer(d18:0/14:0)	835.6021	3.4 × 10^−7^	0.633	13.736	13.103
	LacCer(d18:0/16:0)	863.6334	7.7 × 10^−4^	14.835	17.061	2.226
	LacCer(d18:0/18:0)	891.6647	3.7 × 10^−2^	2.873	15.061	12.188
	LacCer(d18:0/24:0)	975.7586	1.4 × 10^−7^	0.633	15.982	15.349
	LacCer(d18:0/24:1)	973.7429	2.9 × 10^−4^	14.684	17.590	2.905
Prenol Lipids	Loroxanthin ester	764.5744	1.6 × 10^−7^	0.633	15.808	15.175
Fatty Acyls	N-arachidonoyl tyrosine	467.3036	2.5 × 10^−2^	2.701	16.869	14.167
	N-oleoyl taurine	389.2600	2.5 × 10^−7^	0.536	17.091	16.555
Glycerophospholipids	PE(12:0/18:0)	663.4839	4.1 × 10^−2^	16.210	14.996	−1.214
	PE(12:0/20:0)	691.5152	1.3 × 10^−2^	17.396	16.029	−1.367
	PE(14:1(9Z)/22:1(11Z))	743.5465	3.8 × 10^−7^	15.602	0.505	−15.097
	PE(17:1(9Z)/20:3(8Z,11Z,14Z))	753.5309	4.8 × 10^−3^	13.579	14.973	1.394
	PE(18:0/0:0)	481.3168	8.4 × 10^−4^	14.884	17.881	2.997
	PE(18:1(9Z)/22:1(11Z))	799.6091	1.2 × 10^−2^	17.703	16.534	−1.170
	PE(19:0/0:0)	495.3325	5.3 × 10^−3^	18.065	20.749	2.684
	PE(20:1(13E)/20:0)	801.6248	2.2 × 10^−2^	15.342	14.079	−1.263
	PE(20:2(11Z,14Z)/18:3(6Z,9Z,12Z))	765.5309	7.5 × 10^−7^	0.536	16.458	15.922
	PE(20:2(11Z,14Z)/22:0)	827.6404	1.0 × 10^−2^	16.269	14.160	−2.109
	PE(20:3(8Z,11Z,14Z)/22:1(11Z))	823.6091	2.3 × 10^−7^	15.182	16.273	1.092
	PE(22:0/12:0)	719.5465	4.5 × 10^−2^	21.434	20.365	−1.070
	PE(O-16:0/18:3(9Z,12Z,15Z))	699.5203	1.4 × 10^−7^	0.633	18.848	18.215
	PE(O-18:0/14:1(9Z))	675.5203	1.7 × 10^−3^	17.999	15.761	−2.238
	PE(O-18:0/18:4(6Z,9Z,12Z,15Z))	725.5359	2.4 × 10^−3^	17.094	19.268	2.175
	PE(O-18:0/20:4(5Z,8Z,11Z,14Z))	753.5672	3.6 × 10^−3^	16.267	18.210	1.943
	PE(O-20:0/20:2(11Z,14Z))	785.6298	1.1 × 10^−2^	16.238	14.953	−1.285
	PE(O-20:0/22:4(7Z,10Z,13Z,16Z))	809.6298	3.9 × 10^−7^	0.633	13.987	13.354
	PG(15:0/17:1(9Z))	720.4941	2.9 × 10^−3^	0.536	17.568	17.033
	PG(15:1(9Z)/18:4(6Z,9Z,12Z,15Z))	726.4472	6.4 × 10^−6^	13.761	1.084	−12.677
	PG(16:0/22:6(4Z,7Z,10Z,13Z,16Z,19Z))	794.5098	3.4 × 10^−2^	14.952	16.088	1.136
	PG(16:1(9Z)/20:3(8Z,11Z,14Z))	770.5098	4.8 × 10^−2^	3.536	14.294	10.758
	PG(18:3(6Z,9Z,12Z)/20:1(11Z))	798.5411	3.5 × 10^−4^	2.477	14.774	12.297
	PI(13:0/17:0)	782.4945	5.8 × 10^−7^	0.633	13.985	13.353
	PS(14:0/20:3(8Z,11Z,14Z))	757.4894	9.6 × 10^−6^	14.625	1.881	−12.745
	PS(20:3(8Z,11Z,14Z)/22:0)	869.6146	3.4 × 10^−2^	15.945	17.142	1.197
	PS(21:0/22:4(7Z,10Z,13Z,16Z))	881.6146	6.1 × 10^−3^	15.964	14.599	−1.365
	PS(22:0/22:4(7Z,10Z,13Z,16Z))	895.6302	5.2 × 10^−4^	0.633	12.455	11.822
	PS(O-16:0/14:1(9Z))	691.4788	9.3 × 10^−7^	15.318	0.505	−14.814
	PS(O-20:0/21:0)	847.6666	5.2 × 10^−4^	15.863	18.837	2.974
	PS(P-20:0/17:2(9Z,12Z))	785.5571	3.6 × 10^−6^	14.488	1.084	−13.405

## Data Availability

Data are contained within the article and Supplementary Materials.

## Supplementary Materials

The following supporting information can be downloaded at: https://www.mdpi.com/article/10.3390/ijms25084285/s1.
